# Onset and Evolution of Southern Annular Mode-Like Changes at Centennial Timescale

**DOI:** 10.1038/s41598-018-21836-6

**Published:** 2018-02-22

**Authors:** P. I. Moreno, I. Vilanova, R. Villa-Martínez, R. B. Dunbar, D. A. Mucciarone, M. R. Kaplan, R. D. Garreaud, M. Rojas, C. M. Moy, R. De Pol-Holz, F. Lambert

**Affiliations:** 1 Departamento de Ciencias Ecológicas, Universidad de Chile, Santiago, Chile; 2 CONICET-Museo Argentino de Ciencias Naturales, Buenos Aires, Argentina; 3 GAIA-Antártica, Universidad de Magallanes, Punta Arenas, Chile; 4 School of Earth, Energy & Environmental Sciences, Stanford University, Palo Alto, CA USA; 5 Geochemistry, Lamont-Doherty Earth Observatory of Columbia University, Palisades, NY USA; 6 Departamento de Geofísica, Universidad de Chile, Santiago, Chile; 7 Department of Geology, University of Otago, Dunedin, New Zealand; 8 Departamento de Geografía Física, Pontificia Universidad Católica de Chile, Santiago, Chile

## Abstract

The Southern Westerly Winds (SWW) are the surface expression of geostrophic winds that encircle the southern mid-latitudes. In conjunction with the Southern Ocean, they establish a coupled system that not only controls climate in the southern third of the world, but is also closely connected to the position of the Intertropical Convergence Zone and CO_2_ degassing from the deep ocean. Paradoxically, little is known about their behavior since the last ice age and relationships with mid-latitude glacier history and tropical climate variability. Here we present a lake sediment record from Chilean Patagonia (51°S) that reveals fluctuations of the low-level SWW at mid-latitudes, including strong westerlies during the Antarctic Cold Reversal, anomalously low intensity during the early Holocene, which was unfavorable for glacier growth, and strong SWW since ~7.5 ka. We detect nine positive Southern Annular Mode-like events at centennial timescale since ~5.8 ka that alternate with cold/wet intervals favorable for glacier expansions (Neoglaciations) in southern Patagonia. The correspondence of key features of mid-latitude atmospheric circulation with shifts in tropical climate since ~10 ka suggests that coherent climatic shifts in these regions have driven climate change in vast sectors of the Southern Hemisphere at centennial and millennial timescales.

## Introduction

The younger half of the current interglacial, the Holocene epoch, has witnessed the onset of extensive farming, the rise and fall of human civilizations, large-scale anthropogenic impacts on the landscape^1–3^, rapid climate changes^4^ and a recent enhancement of climate variability^5^, forming the backdrop for understanding future climate change during the Anthropocene^6^. Paleoclimate records from tropical latitudes reveal changes in the spatial and temporal modes of El Niño Southern Oscillation (ENSO) variability^5^ over the last ~5000 years, along with insolation-driven shifts in the position of the Intertropical Convergence Zone^7^ and monsoons^8–10^. In contrast to these intensely studied regions, the southern extra-tropics are one of the least understood despite the presence of the SWW, a highly influential component of the hemispheric and global climate systems. The SWW propel western boundary currents, delivery of moisture from the ocean to the southern continents, the Antarctic circumpolar current, upwelling and productivity changes in the Southern Ocean (SO), and degassing of deep ocean waters at high southern latitudes^11^. In southern South America, paleoclimate records indicate renewed glacial activity since the mid-Holocene (~6 ka, ka: 1000 calendar years before 1950 CE). This phenomenon, known as Neoglaciations^12^, paradoxically occurred over an interval of sustained increases in atmospheric CO_2_ concentrations^13^ and in southern mid-latitude summer insolation. The precise timing and extent of Holocene glacier behavior has varied in the literature owing to chronologic and preservation heterogeneities, including reliance on limiting ^14^C ages, among studied regions^14,15^ and, possibly, to differences in the temporal and spatial response to climatic events.

Southern South America (Patagonia, between 40° and 56°S) is the only continuous continental landmass that intersects the core of the SWW, extending south into the SO at the latitude of the Drake Passage. In current climate and during the summer semester, the SWW organize in a circumpolar belt with its core between 50 and 55°S. Southern Annular Mode (SAM)-related variability exhibits positive (negative) correlations to the south (north) of the SWW core^16^. Thus, during SAM positive phases there is a poleward expansion of the band of stronger westerlies – affecting the Antarctic periphery – while the winds tend to weaken at mid-latitudes (ca. between 40 and 50°S). The opposite behavior occurs during the SAM negative phases.

**Figure 1:**
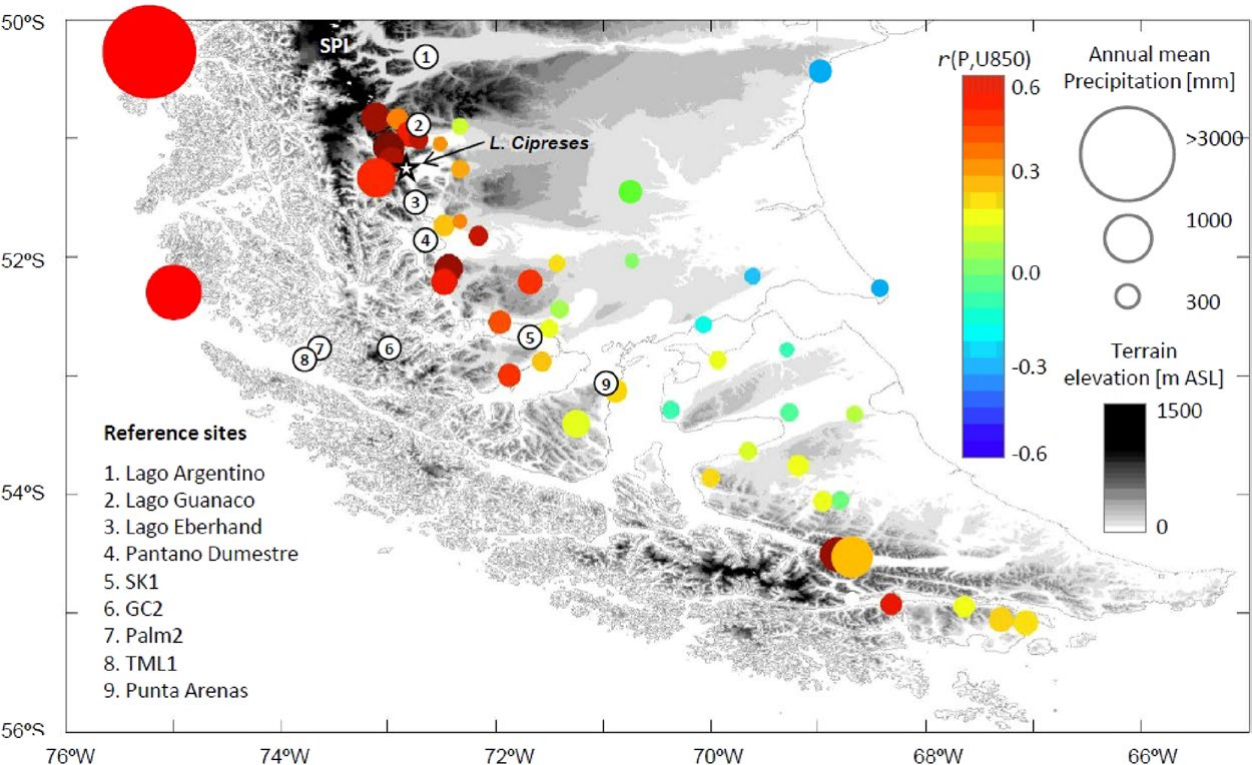
Relationship between low-level zonal wind and precipitation over southern Patagonia at interannual timescales. Each circle is located over a rain gauge with more than 10 years of data superimposed on a topographic map (gray scale at right). Circle size represents the annual mean precipitation according to the scale in the far right, circle color indicates the correlation coefficient between the time series of annually accumulated precipitation and the annual mean 850 hPa zonal wind (from NCEP-NCAR reanalysis) bi-linearly interpolated to the station location. The locations of Lago Cipreses and other reference sites are also indicated. These station-based results are in broad agreement with previous findings based in gridded datasets^17,18^ but provide a finer-scale view of the zonal wind control on precipitation in southern Patagonia. For instance, the stations with red colors near Lago Cipreses indicate that stronger (weaker) than average SWW leads to above (below) average precipitation, even 10–20 kilometers east of the Andes divide, likely as a result of the spillover effect (e.g.^50^).

Throughout the year baroclinic storms embedded in the SWW deliver the precipitation that sustains mountain glaciers, icefields and temperate rainforests along western Patagonia. The Andes Cordillera strongly perturbs these storms: forced ascent of air masses along the windward side of the mountains greatly enhances precipitation in western Patagonia, accompanied by spillover across the Andes and downstream subsidence, generating a marked rain shadow that extends across much of extra-Andean Patagonia^17^. Precipitation over the mid-latitude Pacific Ocean is positively correlated with the intensity of the westerly flow which, in turn, is a proxy of storm growth rate^18^. The correlation increases toward the western coast of South America because of the aforementioned orographic effect. Even locations 50–70 km to the east of the Andean divide show a positive correlation between precipitation and zonal wind (at timescales from daily to annual and longer) (Fig. 1). Because zonal wind over Patagonia is in turn modulated by SAM, this mode of variability exerts an important influence in the form of negative/positive anomalies in precipitation and temperature during the summer months, associated with positive/negative phases of SAM respectively. ENSO also exhibits strong negative correlations with precipitation anomalies in northwestern Patagonia (between 38° and 42°S), a relationship that fades and virtually disappears in weather stations located south of 45°S^19^.

Knowledge of the history of the SWW at millennial and centennial scales remains rudimentary because of the paucity of detailed records and precise chronologies from the southern mid-latitudes. The few available records from southwestern Patagonia provide divergent views and – at times – conflicting interpretations, highlighting the need for additional sites with adequate detail, sensitivity and chronologic precision from constant depositional environments. One important item of discussion is whether the SWW influence was anomalously high^20^ or low^21^ in southwestern Patagonia (between 51° and 53°S) during the early Holocene (between 10.5 and 7.5 ka) and, therefore, for deciphering whether the core of the SWW shifted southward^20^ or whether its strength diminished below modern values^21^ over this interval in the southern hemisphere. Resolving this divergence is important and the strategic location of southwestern Patagonia can be used to test hypotheses stressing the role of changes in the position/intensity of the SWW at the critical latitude of the Drake Passage on variations in SO upwelling, ocean productivity and ventilation of deep-water CO_2_. Recently, we identified SAM-like changes at centennial timescales during the last 3000 years based on a lake sedimentary record from Lago Cipreses (LC)^22^ in southern Chile (Fig. 1). In this study we extend the high-resolution analysis from LC to examine past changes in the SWW since local deglaciation (Supplementary Figures 1 and 2; Supplementary Table 1), and their relationships with Holocene records of atmospheric CO_2_ concentrations, ENSO variability in the eastern tropical Pacific, and Neoglacial activity in southern Patagonia.

## Results

**Figure 2:**
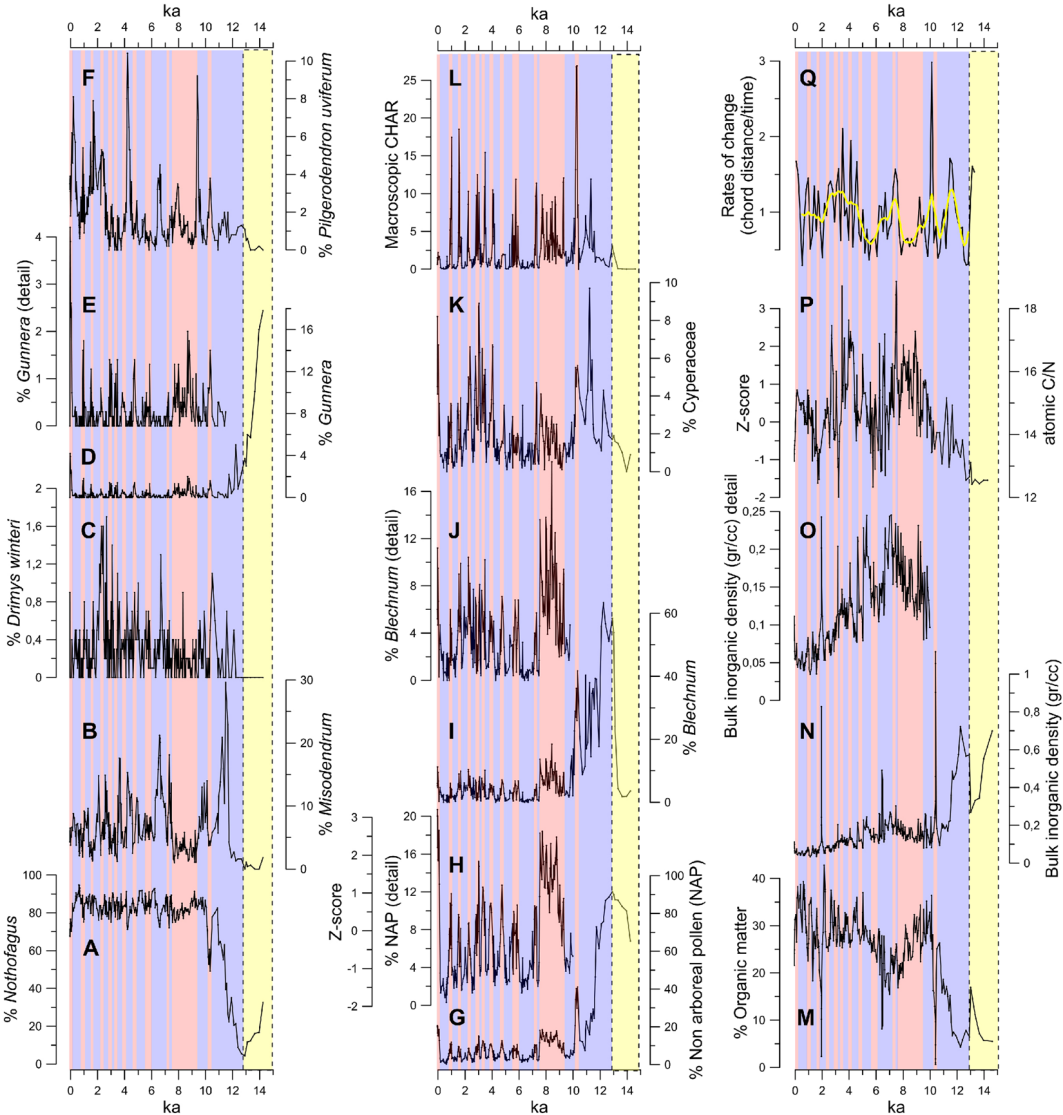
The Lago Cipreses record. The left column indicates percentage variations in arboreal taxa: A = *Nothofagus dombeyi* type; B = *Misodendrum*, a hemiparasite of *Nothofagus*. The presence of this mistletoe attests or the local presence of its host. C = *Drimys winteri*, a relatively hygrophilous tree characteristic of the humid forests of southwestern Patagonia; D = the herb *Gunnera* (possibly *G. magellanica*) and a detailed inset plot (E) to show its Holocene variations; F = *Pilgerodendron uviferum*, a cold-resistant hygrophilous conifer typically found in hyperhumid habitats of Patagonia. The central column shows the sum of terrestrial Non Arboreal Pollen (NAP) in G, and a detailed view (H) of its Holocene variations; the fern *Blechnum* (possibly *B. penna-marina*) in I, and a detailed view (J) of its lower-magnitude Holocene changes; the macrophyte Cyperaceae is shown in K, along with the macroscopic charcoal accumulation rates (CHAR) in L. The right column shows the percent organic matter of the sediments (M), along with the bulk inorganic density (N), and a detail to allow visualization of relatively low-magnitude variations during the Holocene (O). The bulk sedimentary organic matter C/N ratio (P) is expressed in its original units and standardized units in the z-scored secondary scale. The black curve in Q shows the rates-of-change parameter along with a weighted running mean in yellow. The red vertical rectangles indicate dry/warm phases which alternate with cold/humid phases depicted as blue vertical rectangles. The yellow vertical rectangles with dashed black lines denote an extreme cold/wet interval correlative of the Antarctic Cold Reversal.

The LC record suggests a virtually treeless landscape with muted fire activity dominated by cold-resistant herbs and shrubs (Poaceae, Ericaceae, Asteraceae, *Acaena*) between 14.6 and 12.7 ka commonly found in modern high Andean environments and the forest-steppe ecotone. This assemblage also includes herbs, ferns and trees (*Gunnera, Blechnum*, traces of *Pilgerodendron uviferum, Drimys winteri*) (Fig. 2; Supplementary Figure 3) characteristic of hyperhumid sectors along the Pacific coast and moist Andean areas, suggesting cold and humid conditions between 14.6 and 12.7 ka. *Nothofagus* abundance increased rapidly at 12.7 ka accompanied by its hemiparasite *Misodendrum*, and led to the establishment of closed-canopy Magellanic forests (arboreal pollen >90%) by 11 ka (Fig. 2, Supplementary Figures 1 and 3), concomitant with a sustained rise in the organic content of lake sediments, bulk sedimentary organic matter C/N ratios, and a decline in open-ground ferns. This shift suggests gradual warming under humid conditions between 12.7 and 11 ka. Dense temperate forests have dominated the landscape near LC since 11 ka with millennial and centennial variations that we discuss in the following paragraphs.

**Figure 3:**
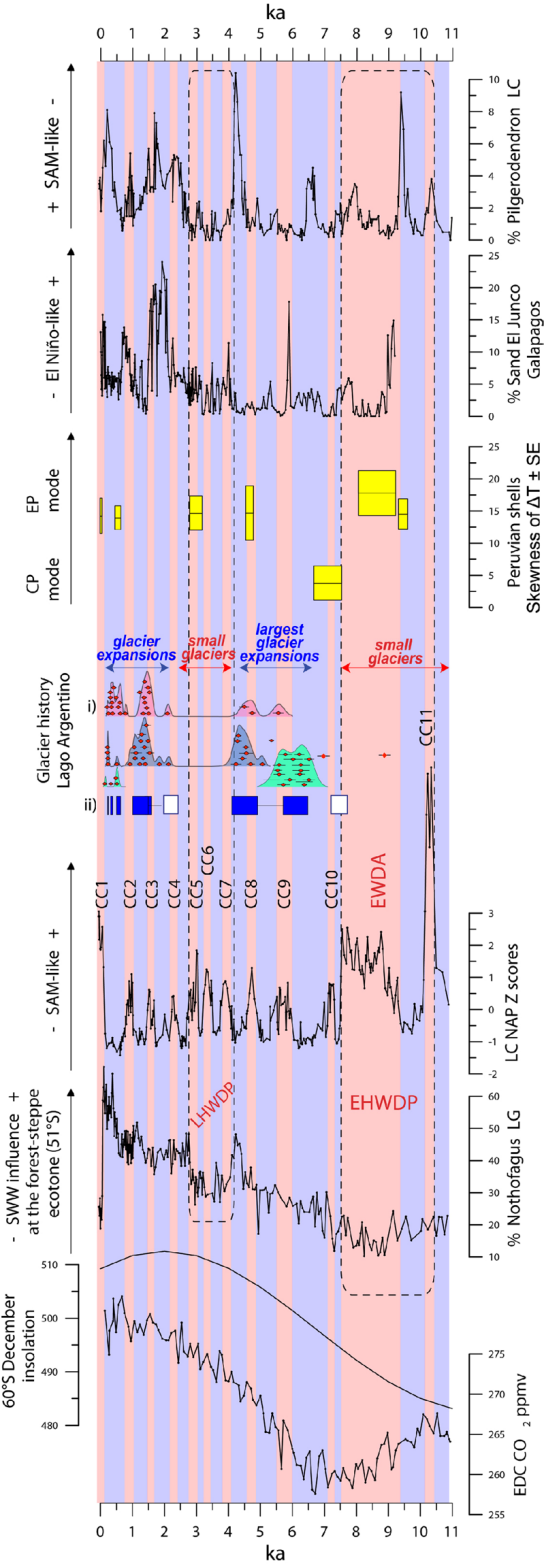
Southern Hemisphere climate comparisons. We compare the last 11,000 years of the standardized Lago Cipreses Non Arboreal Pollen curve (LC NAP)(D) with the the EPICA Dome C record of atmospheric^13^ CO_2_ (A); the December insolation at 60°S (B); the % *Nothofagus* abundance in Lago Guanaco (LG)^21^ (C), a proxy for precipitation of SWW origin in the Torres del Paine area, downwind from LC; a summary of the cosmogenic and radiocarbon constraints (E) on Patagonian glacier history in the Lago Argentino sector^45,46^, we show ^10^Be ages (±1σ), summed probability density plots of all the ages (i.e., camel humps), and mean moraine ages (blue and white rectangles) based on published ^10^Be and ^14^C data. The ‘camel humps’ essentially reflect times of moraine formation and hence glacier expansion relative to today. (F) Shows the skewness of delta temperature (±standard error) from Peruvian shells interpreted^5^ as a proxy for Eastern Pacific (EP, values > 0) or Central Pacific (CP, values < 0) modes in El Niño regime; (G) the % sand record from lake El Junco in Galapagos^38^ (G), a proxy for El Niño activity in the eastern equatorial Pacific; and (H) the % *Pilgerodendron* abundace in the LC record in its original units and standardized units in the z-scored secondary scale, which we interpret as a proxy for negative SAM-like conditions. Also shown are the numbered Cipreses cycles (CC), the Late Holocene Warm Dry Period (LHWDP, bounded by a dashed rectangle), the Extended Warm Dry Anomaly (EWDA), and the Early Holocene Warm Dry Period (EHWDP, bounded by a dashed rectangle). The Lago Argentino record in southern Patagonia includes data for three sectors^13,44^: Herminita Península-Brazo Upsala (pink); Lago Pearson (blue), and Lago Frías (green). We show (i) individual ^10^Be ages (in red, ± 1 σ) along with summed relative probability distributions, with respective colors. The ‘camel humps’ in essence reflect expanded glaciers; (ii) as rectangles, mean ^10^Be ages for moraines (blue, ±1 standard deviation, if n > 4 ages) as well as glacier expansions only dated bylimiting^14^C-dated ranges^13^ (white). Other less well-dated moraine limits (if n < 4 ^10^Be ages) are shown by black horizontal lines (~5.5-5 and 1.4–1.1ka).

The non-arboreal pollen (NAP) sum captures the variability of the LC pollen record over the interval dominated by closed-canopy forests (Figs 2, 3, Supplementary Figures 5 and 6 and associated discussion), and provides a useful parameter for establishing comparisons with other records. The standardized LC NAP record reveals an ~1800-year long, large-magnitude and uniformly positive anomaly between 9.3 and 7.5 ka, driven mainly by increases in Poaceae, Ericaceae and Asteraceae (Supplementary Figure 3). This signal is coeval with high abundance of ferns, littoral vegetation (Cyperaceae) and macroscopic charcoal, along with a steady rise in bulk inorganic density, a persistent decline in percent organic matter and a positive anomaly in C/N ratios (Fig. 2). Altogether, the data suggest that discontinuities in the forest canopy allowed the proliferation of understory shrubs, herbs and ferns, contemporaneous with centripetal shifts of Cyperaceae, local fire occurrence, increased contribution of terrestrial organic matter in the lake and increased runoff/internal reworking of sediments driven by a low lake level stand. We refer to this interval with the informal term Extended Warm/Dry Anomaly (EWDA).

We detect 11 positive anomalies in the LC NAP lasting 200 ± 60 years (mean ± 1σ), which correspond to warm/dry intervals that we named Cipreses cycles^22^ (CC) 1 through 11 (Fig. 3, Supplementary Figure 4, Supplementary Table 2) since 10.3 ka. CC11 (between 10.3 and 10.1 ka) and CC1 (between 1800 CE and the present) constitute the largest-magnitude events which contrast with the more subdued CC2 through 10 (Figs 2 and 3; Supplementary Figure 4). Plausible explanations for these differences involve: (i) the synergistic effect of volcanic and fire disturbance with a warm/dry anomaly, considering that CC11 occurs immediately after the deposition of a 4-cm thick tephra (Supplementary Figure 1); and (ii) the direct (disturbance by fire) and indirect (atmospheric circulation changes) effect of human perturbations during CC1, which promoted vegetation changes in the LC sector since the mid-20^th^ and late 19^th^ centuries^22^, respectively. We interpret the CC as positive SAM-like states^22^, which alternate with cool/wet centennial intervals that we interpret as negative SAM-like states lasting 500 ± 200 years (Supplementary Table 2). We observe that the cold-resistant hygrophilous conifer *Pilgerodendron uviferum*^23^ increases gradually during negative-SAM states, and declines rapidly at the onset of positive-SAM conditions (Figs 2 and 3). This behavior can be explained by its slow-growth and high sensitivity to summer precipitation levels^23^. Tree-ring studies have described negative correlations in the growth rate of this conifer and SAM in northwestern Patagonia^24^.

The interval between 10.5 and 7.5 ka is dominated by positive anomalies in LC NAP (EWDA + CC11), separated by a cold/wet reversal lasting 700 years (Fig. 3). We refer to this 3000-year long interval with the informal term Early Holocene Warm/Dry Period (EHWDP), which was terminated by a 1700-year extended cold/wet period between 7.5 and 5.8 ka, punctuated by a brief reversal (CC10) (Supplementary Figure 4). This was followed by a highly variable interval since 5.8 ka with nine CC and a distinct clustering of large-magnitude positive SAM-like anomalies between 4 and 2.7 ka (CC5, CC6, CC7) we term, informally, the Late Holocene Warm/Dry Period (LHWDP) (Fig. 3). Predominantly cold/wet conditions (negative SAM-like) ensued between 2.7 and 0.2 ka, overprinted by CC2, CC3 and CC4. We note conspicuous increases of *Pilgerodendron uviferum* between 2.7 and 1.5 ka and between 0.6 and 0.2 ka suggesting peak cold/wet conditions (Fig. 2) during negative SAM-like states.

The rates-of-change parameter (ROC), sensitive to the magnitude and rapidity of changes in the terrestrial vegetation^25^, reveals multiple peaks in the LC record indicating that several transitions in the stratigraphy were abrupt. ROC indicates prominent maxima associated with: (i) the *Nothofagus* rise by 11.7 ka, (ii) climatic and disturbance-driven changes during CC11 (10.1 ka), (iii) an abrupt end of EHWDP, and (iv) the last 4600 years (CC1 through 8) (Fig. 2).

Bulk sedimentary organic matter C/N ratios are low in the oldest interval of the LC section and exhibit a sustained rise leading to a positive anomaly between 10 and 7 ka, suggesting peak influence of terrestrial organic matter during the EWDA (Fig. 2). A reversal into negative anomalies ensued, followed by another rise to peak positive anomalies during the LHWDP and marked variability around the mean until the present. We note that the multi-millennial variations in C/N and LC NAP are largely coincident (Fig. 2), suggesting that extended warm/dry conditions drove lake-level lowering that increasing the delivery of terrestrial organic matter into LC.

**Figure 4:**
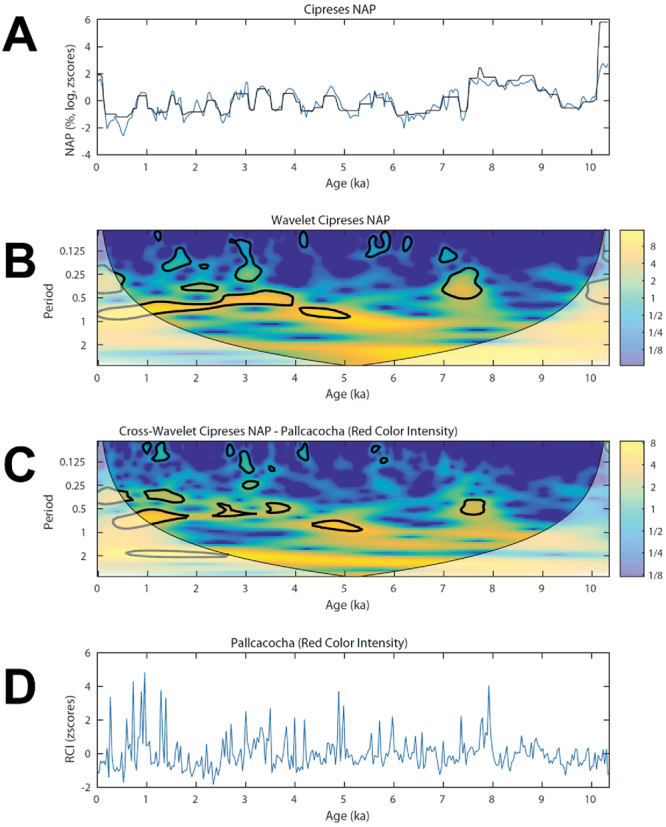
(**A**) Interpolated z-scored Lago Cipreses (LC) Non arboreal pollen (NAP) record in linear (black line) and log scale (blue) line. (**B**) Wavelet power spectrum of the log-transformed LC NAP data using a Morlet wavelet^51^, the contoured areas are significant at the 95% level. The shaded areas outside the cone of influence represent periodicities that are subjected to edge effects. (**C**) Cross wavelet transform of the LC NAP data and the red-intensity data from Laguna Pallcacocha^39^ (shown in **D**). The cross-wavelet analysis reveals high common power during the early-to-late Holocene transition at on the multi-centennial bandwidth between 8–7.5 ka and during the most recent ~5500 years.

Wavelet analysis of the LC NAP data shows (Fig. 4) inception of sub-millennial periodicities at ~5.8 ka, following the stable EHWDP and the beginning of the highly variable interval with the various CCs. Statistically significant periodicities during this interval range between 500 and 1000 years, which are consistent with the sum of ~700 years of the warm/dry and cold/wet states detected by the regime-shift algorithm^26^ (Supplementary Figure 4). The wavelet detects lower-amplitude periodicities between 4 and 2.5 ka, contemporaneous with the cluster of closely spaced large-magnitude CCs during the LHWDP.

## Discussion

Closed-canopy *Nothofagus* forests established near Lago Cipreses at 11 ka and have persisted since then with variations at millennial and centennial timescales. The LC record shows an interval of reduced precipitation brought by weak SWW impinging southwestern Patagonia during the early Holocene (EHWDP, between 10.5 and 7.5 ka), followed by a multi-millennial rise in precipitation related to stronger SWW. These findings match terrestrial records from northwestern (42°S)^21,27,28^, central-west^29,30^ (46°S), southwestern^21,31^ Patagonia (51°S) and Staten Island (54°S)^32^ suggesting a widespread negative anomaly in SWW influence in western South America between 42° and 54°S. Exceptions to this regional pattern are the results^20^ from sites 6 and 7 (Fig. 1), which have been interpreted as a prominent positive anomaly in SWW influence during the early Holocene. Our conclusions are consistent with Tasmanian records^33,34^ (42°S), located ~9000 km west of LC on the opposite side of the South Pacific Ocean, suggesting a zonally symmetric behavior of the SWW at multi-millennial timescale. Because the SWW minimum correlates with a conspicuous decline in atmospheric CO_2_ during the early Holocene, and its subsequent strengthening with a rising CO_2_ trend that started at ~7 ka^13^ (Fig. 3), we posit that variations in SWW strength drove SO overturning and contributed to atmospheric CO_2_ changes at multi-millennial scale during the Holocene^21,35^, i.e. weak SWW flow between 10.5 and 7.5 ka at the latitude of the Drake Passage (between 56° and 61°S) reduced the upwelling of CO_2_-enriched deep waters in the SO concurrent with a multi-millennial halt/reversal in the CO_2_ rise^21,35^; stronger SWW and enhanced surface wind stress on the SO starting at 7.5 ka intensified deep water convection and release of CO_2_ to the atmosphere^11^. The SWW contribution to changes in the Holocene atmospheric carbon inventory is complementary to hypotheses^36^ stressing changes in terrestrial biomass and ocean carbonate compensation.

Within the strong SWW interval we observe a cold/wet anomaly between 7.5 and 5.8 ka, followed by the onset and intensification of centennial-scale variability. Warm/dry SAM-positive-like events (CCs) lasting 200 ± 60 years alternated with 500 ± 200 year-long cold/wet SAM-negative-like phases over the last 11,000 years, with conspicuous clustering of events over the last 5800 years and during the 4–2.7 ka interval (LHWDP) (Fig. 3). The most recent CCs in the LC record correspond in timing with the Medieval Climate Anomaly^37^ (CC2) and the Anthropocene^6^ (CC1) separated by an extended cold/wet SAM-negative-like interval, contemporaneous with the Little Ice Age^22^ in the Northern Hemisphere. This correspondence in timing and direction of climate change with Northern Hemisphere records suggests interhemispheric correlation of centennial-scale events over the last millennium, apparently mediated through changes in the position and intensity of the SWW^22^. Recent studies from northwestern Patagonia^28^ and western Tasmania^33,34^ identified a similar shift from multi-millennial to sub-millennial-scale variability at ~5.3 ka. Because interannual precipitation and temperature anomalies in those regions are driven both by tropical (ENSO) and extra-tropical (SAM) modes of variability, their distinction remains elusive in stratigraphic records typically featuring temporal resolution of multiple decades. Precipitation anomalies in Southwestern Patagonia, on the other hand, are primarily or exclusively controlled by SAM^19^ providing a clearer source for exploring past changes in extra-tropical climate variability. It appears then that SAM-like variability may have affected the windward-facing sectors of southern mid-latitude landmasses in the most recent half of the Holocene.

Linking our findings into a broader spatial perspective, we observe correspondence of key Holocene events in the LC NAP data and ENSO-sensitive records from the eastern tropical Pacific sector (Fig. 3), namely: (1) the EWDA was contemporaneous with the predominance of an Eastern Pacific (EP) mode in ENSO activity^5^; (2) during the opposite condition (i.e. the extended and relatively homogeneous cold/wet anomaly) ENSO was dominated by a Central Pacific (CP) mode (between 7.5 and 6.7 ka)^5^; (3) peak variability in southwestern Patagonia since 4.6 ka is coeval with warmer sea surface temperature in central coastal Perú and the establishment of the modern ENSO regime (EP mode)^5^; and (4) persistently negative SAM-like conditions in LC (persistently negative anomalies in NAP and high positive anomalies in *Pilgerodendron uviferum*) (Fig. 3) is contemporaneous with peak El Niño activity (between 2–1.3 ka) in Lago El Junco^38^ (located in the Galapagos islands) (Fig. 3) and Laguna Pallcacocha^39^ (located in the Ecuadorian Andes)(Fig. 4). Cross-wavelet analysis of the LC NAP and the latter record reveals high common power on the multi-centennial bandwidth in the younger half of both records, when ENSO and SAM-like variability reached full development (Fig. 4). These tantalizing correspondences in the timing of mode shifts and time-frequency space suggest a common tropical and southern mid-latitude response in the eastern sector of the South Pacific basin. Although SAM variability (i.e., transitions from positive to negative polarity) emerges from internal atmospheric dynamics in the southern mid-latitudes^40^, it is becoming clearer that ENSO and SAM are not fully independent^41,42^. The connection arises, at least partially, because tropical warm SST anomalies during El Niño years force atmospheric Rossby waves that propagate over the Pacific toward high latitudes supporting a negative polarity of SAM (i.e., positive pressure anomalies at higher latitudes). The opposite condition occurs during La Niña years. This is evident as a negative association between Niño3.4 and SAM indices, although the strength of their correlation varies at seasonal and decadal time scales^2,3,43,44^. Through this tropical-extratropical mechanism, augmented ENSO variability can excite more energetic SAM variations, providing a potential link for the near-synchronous birth and millennial-scale structure of ENSO and SAM-like variability after ~5.8 ka.

Our findings also provide insight into past glacier behavior in Patagonia, including during the Neoglacial. Eastward-flowing outlet lobes from the South Patagonian icefield^16^ (SPI) deposited moraines at the head of Lago Argentino (50°30′S) (Fig. 1) multiple times during the Holocene. Recently obtained exposure-ages^45^, which date directly former glacier limits, along with radiocarbon constraints^46^ provide firm data on the timing of Neoglacial events in southwestern Patagonia. Comparison of the LC NAP data with this glacial chronology at Lago Argentino reveals that predominantly cold/wet conditions (negative SAM-like) correspond in timing with the culmination of a glacier advance during the Antarctic Cold Reversal (bracketed between 13 and 12.7 ka^47^) and the largest Neoglacial advances^45^ dated from 6.1 ± 0.4 to 4.5 ± 0.4 ka (Fig. 3, Supplementary Figure 9). Moreover, these glacier expansions were immediately followed by warming at 12.7 ka, 5.8 ka (CC9), and 4 ka (CC7) (Fig. 3). A conspicuous lack of glacier expansions is evident during both the EHWDP and LHWDP, with the latter followed by culmination of expanded glaciers around ~2–2 and 1.5–1 ka, and successive ice margin positions between 0.6–0.2 ka^45,46^ (Fig. 3 and Supplement information). These events also fall within cold/wet intervals and were followed by CC3, CC2 and CC1, respectively (Supplementary Figure 9). We propose that the ACR and Neoglacial advances occurred during centennial negative SAM-like conditions that fueled their growth, and likewise, that CCs (positive SAM-like events) may have been key drivers of glacier recession and maintenance of smaller limits (e.g., similar to present) (Fig. 3), given warm and dry conditions enable ablation and reduced accumulation.

Recent modelling studies explored the role of deep convection in the SO^48^ and fluctuations in Antarctic Ice Sheet discharge^49^ as drivers or amplifiers of Holocene variability. Both mechanisms produced Antarctic sea-surface and surface-air temperature change at centennial timescale anomalies and teleconnections with the southern mid-latitudes and the North Atlantic region, providing potential mechanisms for the generation of climate variability in mid-latitude South America. A missing link in these simulations, however, is the role of centennial-scale changes in the SWW as a potential driver of sensitive components of the climate system, such as SO convection, polar front migration, and subsurface ocean temperature via changes in the latitude and intensity of wind stress. Incorporation of SWW variability at centennial and millennial timescales in these models may lead to significant advances in future simulations.

We propose that changes in the coupled ocean-atmosphere system in the tropical Pacific and Southern Ocean during the early-to-mid Holocene transition (7.5 ka) invigorated gyre circulation in the South Pacific and the SWW, contributed to a rise in atmospheric CO_2_ content, and initiated Neoglaciations in southwestern Patagonia. ENSO- and SAM-like variability at centennial timescales established after ~5.8 ka and reached peak development over the last 4600 years, indicating coherent changes in tropical and extra-tropical modes of variability.

## Electronic supplementary material

Supplementary material
