# Supplementary material for: Onset and Evolution of Southern Annular Mode-Like Changes at Centennial Timescale
Source: Sci Rep. 2018 Feb 22;8:3458. doi: 10.1038/s41598-018-21836-6 (PMC5823851; doi:10.1038/s41598-018-21836-6)
Supplement: Supplementary file 1 — Supplementary material [file 41598_2018_21836_MOESM1_ESM.docx]

Supplementary material on

ONSET AND EVOLUTION OF SOUTHERN ANNULAR MODE-LIKE CHANGES AT CENTENNIAL TIMESCALE

Moreno, P.I.^1*^, Vilanova, I.^2^, Villa-Martínez, R.^3^, Dunbar, R.B.^4^, Mucciarone, D.A.^4^, Kaplan, M.R.^5^, Garreaud, R.D.^6^, Rojas, M.^6^, De Pol-Holz, R.^3^, Lambert, F.^7^

1. Site setting

Lago Cipreses is a small, closed-basin pond located on a bedrock depression along the southwestern portion of Lago Toro (51°17'6.15"S , 72°51'13.48"W, 110 m.a.s.l.), ~85 meters above Lago Toro (25 m.a.s.l.). The site lies in an ice molded sector ~60 km SE of the South Patagonian icefield and ~12 km upstream from the C moraines mapped by Marden^[1](#_ENREF_1)^ at the southern end of Lago Porteño. Marden^[1](#_ENREF_1)^ interpreted these C moraines as a southward expansion of the Lago Toro ice lobe during the Last Glacial Maximum (LGM) on the basis of morphostratigraphic position, and minimum-limiting radiocarbon and tephrochronologic constraints associated with (much younger) glacial deposits located >35 km upstream along the glacier flow line. Solari et al.^[2](#_ENREF_2)^ reported two radiocarbon dates from glaciolacustrine beds distal to moraines B and C along the eastern end of Lago Toro which afford maximum ages of 24 and 21 ka for these moraines, consistent with Marden’s tentative LGM age for these deposits and landforms. The basal date of 14.6 ka from Lago Cipreses constitutes a minimum-limiting age for local ice-free conditions and cessation of glaciolacustrine influence at the site, indicating that the Lago Toro ice lobe had retreated northward from the C moraines located in the southern shore of Lago Toro and associated ice-margin glaciolacustre environments had lowered below 110 m.a.s.l. prior to the onset of organic lacustrine sedimentation in a closed-basin environment in Lago Cipreses. These data imply that major recession (≥12 km, ≥50% of the total length/width of Lago Toro) from the C moraines located south of Lago Toro was achieved prior to the Antarctic Cold Reversal (13-14.6 ka).

2. Methods and results

We retrieved a sedimentary record from Lago Cipreses using a 7.5-cm diameter transparent water-sediment interface corer and a 5-cm diameter square-rod piston Livingstone corer from an anchored coring rig we placed on the deepest sector of Lago Cipreses. We spliced these records based on the presence of a common tephra layer and loss-on-ignition data (see below). The spliced record consists of 365 cm of organic silts with varying amounts of inorganic silts and fine sands that grade into well-sorted medium-to-fine sands in the lower part of the stratigraphy (Supplementary Figure 1). We detect two tephras at 79-81 and 301-304 cm.

The chronology of the LC record is constrained by 15 contiguous ^210^Pb dates[^3^](#_ENREF_3) from the uppermost part of the water sediment interface core and 23 radiocarbon dates on bulk organic sediment distributed throughout the sedimentary record (Supplementary Table 1, Supplementary Figure 1). These dates constitute the basis for a Bayesian age model we developed using Bacon[^4^](#_ENREF_4) (Supplementary Figure 2). The stratigraphy, chronology and age model indicate continuous accumulation of organic lake sediments in Lago Cipreses since 14.6 ka.

We characterized the stratigraphy of the LC record through textural descriptions, digital X radiographs and loss-on-ignition analysis[^5^](#_ENREF_5) on 1-cc samples (Supplementary Figure 1). The atomic C/N ratio was measured on freeze-dried samples we crushed and loaded into silver capsules for bulk elemental analyses. Prior to analysis, sediment samples were acidified with a 6% sulfurous acid solution to remove carbonates. All samples were analyzed at the Stanford University Stable Isotope Laboratory using a Carlo Erba NA1500 Series 2 elemental analyzer.

The pollen and macroscopic charcoal records include 319 contiguous 2-cc samples obtained from 1-cm thick sections. The palynological samples were processed using a standard procedure that included 10% KOH, sieving with a 120-µm mesh, 40% HF and acetolysis^[6](#_ENREF_6)^; the concentrates were mounted in silicon oil 2000 cps and analyzed at 400X magnification using a stereomicroscope. The results are expressed as percentage abundance relative to sums that include all terrestrial taxa (arboreal + non arboreal pollen [NAP]), total pollen (terrestrial + aquatic pollen) and spores (total pollen + spores) (Supplementary Figure 3). We used a regime shift detection tool[^7^](#_ENREF_7) to aid in the recognition of changes in the NAP and allow a more objective definition of the time boundaries for the fluctuations discussed in the text (Supplementary Figure 4). We calculated the rates-of-change parameter to quantify the magnitude and rapidity of changes in the pollen stratigraphy[^8^](#_ENREF_8). This involved calculation of a dissimilarity value (chord distance) between adjacent pollen samples following interpolation at regular 150-year steps, after recalculating sums and percentages on all terrestrial pollen taxa having abundance ≥2%.

The samples for macroscopic charcoal analysis were deflocculated in KOH 10%, rinsed gently to avoid breakage of particles and analyzed at 10X-50X under a stereoscope. We performed time-series analysis of the macroscopic charcoal data to detect local fire events using the CharAnalysis software[^9^](#_ENREF_9), through deconvolution of the peaks signal from slowly varying background abundance. For that purpose, we interpolated samples at the median time step between charcoal levels and subtracted the background component using a lowess robust to outliers smoother with a 1000-yr window width, and calculated locally defined thresholds through the time series to identify statistically significant charcoal peaks (99^th^ percentile of a Gaussian distribution), which we interpret as local fire events.

3. Palynology

*Nothofagus* in this study refers to the palynomorph *Nothofagus dombeyi* type*,* which includes three tree species (*N. betuloides,* *N. pumilio*, *N. antarctica*)[^10^](#_ENREF_10) that occur in different plant communities throughout SW Patagonia, from sea level to the upper treeline and from the hyperhumid Pacific coast to the forest/steppe ecotone east of the Andes. We observe low *Nothofagus* abundance (mean: 13%) between 12.7-14.6 ka followed by a rapid increase we interpret as the initial establishment of a scrubland that transitions into a woodland and culminates with closed-canopy forests that have persisted near Lago Cipreses over the last 11,000 years (mean: 83%) (Supplementary Figure 3). Peak abundance of *Gunnera* strongly suggests high precipitation regime between 12.7-14.6 ka considering its modern distribution in the humid and hyperhumid environments west of the Andean Range, both above and below the modern treeline, and its absence in Patagonian steppe environments. *Gunnera magellanica* commonly occurs in the periphery of glaciers, mantling morainal topography and is one of the early colonizers of recently deglaciated terrains in humid sectors of throughout Patagonia. Low *Nothofagus* and peak abundance of *Gunnera* between 12.7-14.6 ka suggests that precipitation was not a limiting factor for the development of arboreal vegetation, instead we propose that low temperatures and biological/biogeographical constraints inhibited the local proliferation of *Nothofagus* during the Antarctic Cold Reversal in this sector of SW Patagonia.

We performed a PCA to detect the main sources of variability on the LC record. The PCA ordination shows that *Nothofagus,* on one hand, and the non-arboreal pollen (NAP) sum and ferns, on the other hand, plot opposite along PCA axis 1 (56.1%). Cyperaceae and CHAR show similar, equally strong, segregation along PCA axis 2 (23.7%) (Supplementary Figure 4). When constraining the analysis to the interval dominated by *Nothofagus* forests (the last 10,800 years) we observe a separation of *Nothofagus* opposite to a tight association of NAP and ferns and, to a certain extent, Cyperaceae along PCA axis 1 (63.7%). CHAR appears separated from the other variables with a strong contribution to PCA axis 2 (13.9%) (Supplementary Figure 5). Supplementary Figure 6 includes the PCA Axis 1 scores alongside % NAP plotted along the age scale. It is evident from this comparison that PCA Axis 1 is nearly identical to variations in % NAP in the Lago Cipreses record. Altogether, the PCA results validate the usage of NAP, ferns and Cyperaceae as the main sources of variation in the LC pollen record, along with CHAR. We interpret that forest canopy continuity vs fragmentation, lake level fluctuations and fire occurrence have covaried in a coherent and systematic manner through the Holocene. These centennial-scale changes reflect hydroclimate variations akin to the modern functioning of the SAM.

The standardized LC NAP record reveals an 1800-year long, large-magnitude and uniformly positive anomaly between 9.3 and 7.5 ka, driven mainly by increases in Poaceae, Ericaceae and Asteraceae (Supplementary Figure 3). This signal is coeval with high abundance of ferns, littoral vegetation (Cyperaceae) and macroscopic charcoal, along with a steady rise in bulk inorganic density, a persistent decline in percent organic matter and a positive anomaly in C/N ratios (Figure 2). Altogether, the data suggest that discontinuities in the forest canopy allowed the proliferation of understory shrubs, herbs and ferns, contemporaneous with centripetal shifts of Cyperaceae, local fire occurrence, increased contribution of terrestrial organic matter in the lake and increased runoff/internal reworking of sediments driven by a low lake level stand. We refer to this interval with the informal term Extended Warm/Dry Anomaly (EWDA).

The fern *Blechnum* (possibly the species *B. penna-marina*) increased rapidly and achieved a ~57% peak between 12-13 ka, suggesting abundant open-ground and humid upland environments, followed by a decline and persistence at intermediate abundance (30%) until 10.1 ka during the encroachment of *Nothofagus* woodlands and forests, and then declined to values <16% over the last 10,000 years. These variations indicate reduction in the amount of light reaching the floor, the substrate where *B. penna-marina* thrives, in response to increasing degrees of arboreal cover in the landscape. *Misodendrum*, a hemiparasite of *Nothofagus* species, attained a peak during this interval (30% at 11.7 ka), illustrating its dependence on intermediate levels of woodland/forest cover and luminosity. We note synchronous increases in *Blechnum* and NAP at centennial and multi-millennial scales over the last 10,000 years during episodes of partial forest opening, most probably generated by small-scale gaps or scattered clearings.

The conifer *Pilgerodendron uviferum* produces pollen grains with small dispersal capability and its abundance is normally underrepresented in surface samples. On the contrary, the conifer *Podocarpus nubigena* produces large quantities of bisaccate grains prone to long-distance transport by strong SWW. The species *Drimys winteri*, on the other hand, produces small amounts of very large grains released as tetrads that normally are dispersed over short distances and is normally underrepresented in surface samples as well. In view of these considerations we interpret the local presence of the hygrophilous and cold-resistant trees *P. uviferum* and *D. winteri* as indicative of local presence of a humid temperate Magellanic forest since 11 ka, most probably dominated by *Nothofagus betuloides* accompanied by *P. nubigena*. We note prominent peaks in *P. uviferum* at 4.3 and 9.4 ka in the context of cold/wet intervals just prior to the LHWDP and a reversal within the EHWDP, respectively. This was followed by a conspicuous decline between 2.7-4 ka, coeval with the LHWDP, and a prominent increase between 1.5-2.7 ka punctuated by a reversal at ~2 ka (CC4). *P. uviferum* declined again and rose to a maximum between 0.2-0.6 ka during a cold/wet interval that correlates with the Little Ice Age[^3^](#_ENREF_3).

We interpret the large magnitude of vegetation and fire-regime changes during CC11 as the result of climatic and non-climatic forcing of terrestrial ecosystem change, considering that CC11 immediately overlies a 4-cm tephra deposited at 10.4 ka. These results suggest a synergistic effect of volcanic disturbance and warm/dry conditions, most likely mediated by fire activity and changes in physical/chemical properties of soils that caused an abrupt and temporary shift in the pollen assemblage. We note that the deposition of a 3-cm thick tephra at 1.9 ka, during a cold/wet interval, is associated with a modest and ephemeral increase in NAP and no impact on littoral vegetation or macroscopic charcoal, suggesting that the magnitude of terrestrial and aquatic ecosystem responses to volcanic disturbance are modulated by the mean climatic state, as suggested by recent studies in the temperate rainforest region of northwestern Patagonia [^11^](#_ENREF_11)^,^[^12^](#_ENREF_12).

4. Fire history

The Charcoal Accumulation Rate (CHAR) record from Lago Cipreses shows increases associated with positive anomalies in the LC NAP and declines during negative anomalies (Supplementary Figure 7). We conducted CharAnalysis^[9](#_ENREF_9)^ to identify local fire events, defined as statistically significant CHAR peaks, and examine the temporal structure of local fire. The analysis reveals 25 local events, the majority of which correspond in timing with positive anomalies in the standardized LC NAP record, the establishment of closed-canopy forests between 10-11.6 ka and warming at 12.7 ka, suggesting that temperature rise was the primary control on fire occurrence in the vicinity of Lago Cipreses. The largest-magnitude peaks correspond in timing with CC2, CC3 and CC11 and occur at times of average fire frequency, whereas intervals of peak fire frequency (3, 5.7, 8-9 ka) feature relatively low peak magnitudes (Supplementary Figure 3).

5. Time series analysis of the LC NAP record

We performed wavelet analysis on the log-transformed LC-NAP z-score data using the Cross Wavelet and Wavelet Coherence toolbox for MATLAB[^13^](#_ENREF_13). The log-transformed LC NAP data proved adequate for this analysis considering their normal distribution (Supplementary Figure 8). The power spectrum shows (Figure 3) a clear inception of sub-millennial periodicities around 6 ka, following the stable EHWDP and the beginning of the highly variable interval with the various CCs. The periodicities detected in that interval are between 500 and 1000 years, which is consistent with the sum of 700 years of the warm and cold periods detected by the regime-shift algorithm (Supplementary Figure 8). Furthermore, there is a clear interval between 2.5-4 ka with lower periodicities that corresponds to the LHWDP mentioned in the main text with higher frequency changes between the warm/dry and cold/wet states.

6. Holocene glacier history

We include glacier histories from the Lago Argentino area, just north of Lago Cipreses (Figure 1), recently summarized and discussed by Strelin et al.[^14^](#_ENREF_14), which also incorporated prior investigations (e.g.[^15^](#_ENREF_15)^,^[^16^](#_ENREF_16) ), and Kaplan et al.[^17^](#_ENREF_17).

Several salient aspects of the glacier history dovetail with our findings. These include:

1) Times of reduced ice extent, when glaciers were similar to or perhaps even behind their present margins, correspond to warm/dry phases (Figure 4, Supplementary Figure 9). Most notable, there are two main intervals of small glaciers, which correspond one-to-one with the EHWDP and LHWDP. These warm/dry periods not only contain distinct gaps in observed moraines, but, in the Agassiz Este Valley Strelin et al.[^14^](#_ENREF_14) specifically dated forest material that lived near the present ice margin during the EHWDP. The dated reworked wood documents that forests lived near the margin which may have even been slightly less extensive[^14^](#_ENREF_14) . We posit that warm and dry conditions during the EHWDP and LHWDP were, simply put, not conducive to positive mass balance of glaciers.

2) Times of repeated glacier expansions or readvances (e.g., cross cutting relations, stratigraphic evidence) correspond to cold/wet phases (Supplementary Figure 8). The most well-dated moraine building events occurred at 6120±390 (n=13 ^10^Be ages), 4450±220 (n= 7), ~1450 or 1,410±110 (n=18), 360±30 (n=5), and 240±20 (n=8) years ago. In addition, ^10^Be ages (supported by ^14^C data) indicate that glaciers were generally expanded from ~6000 to ~4800 (n=5), at ~2.2 ka (n=2), and from ~1400 to 1000 years ago (n=9) and 600-500 years ago (n=7). The uncertainties include propagation of an error for the production used, to facilitate comparison with ^14^C based chronologies. For Figure 3 and the associated discussion, we do not subtract ~60 years from the moraine ages, as ^14^C years are provided relative to 1950 whereas the cosmogenic ages are provided relative to the year of collection (i.e., ~2010-1950 = 60)[^17^](#_ENREF_17). This small difference has no effect on our inferences given the resolution of the figure, for example. If 60 years is subtracted, the moraine ages above are ~6060±390 (n=13), 4,390±220 (n= 7), ~1,340 or 1,350±110 (n=18), 300±30 (n=5), and 180±20.

Regarding correspondence between the Cipreses record and the glacier history we note the following: (i) individual ^10^Be age uncertainties are routinely ~ 5% or better[^17^](#_ENREF_17) prior to ~1-2 ka, producing absolute errors that typically fall within 100 to 300 years; thus, in general, precise one-to-one correlation between dated moraines and the onset and end of a CC event cannot be done with confidence (Figure 4). (ii) The events dated between ~6 and 4.4 ka are the most expansive advance during the Holocene, and they followed a prolonged warm and dry period in the early Holocene (EHWDP), when evidence indicates margins were close to or at present (see above). We note that the ~6 and ~4.5 ka advances occurred during a predominantly cold and wet multi-millennial interval during the middle Holocene, and interpret that CC9 and CC8 may have driven their recession. (iii) Toward younger timescales individual ^10^Be uncertainties are smaller in an absolute sense, within a few decades (e.g., 300±30, and 180±20). When absolute errors are within a few decades, we indeed note the excellent correlation between ^14^C-^10^Be dated glacier (both ^14^C and ^10^Be) and CC events, which occurs during the last 2000 years, namely: (a) the last three dated glacier events fall precisely within the inter CC1 - CC2 period and (b) the ~1-1.4 ka set of advances (Figure 4) lie between the inter CC2-CC3 period. Strelin et al. dated an event between ~2-2.5 ka, which is supported by one ^10^Be age obtained[^17^](#_ENREF_17). Hence, for events older than 2 ka, we can no longer confidently match precisely one-to-one the glacier history with the onset and end of CC events.

Tephra

Tephra

Supplementary Figure 1. Stratigraphic column and results of the loss-on-ignition analysis of the Lago Cipreses spliced record (IBD= inorganic bulk density). The panel on the right presents a detail of the IBD data, the high values associated with a tephra were truncated to permit visualization of lower-magnitude fluctuations.

Supplementary Figure 2. Calibrated probability age distributions from Lago Cipreses (blue zones), also shown is the Bayesian age calculated on the calibrated radiocarbon-age data and the ^210^Pb chronology for the upper part of the record. The dashed lines establish the 95% confidence limits of the modeled ages.

Supplementary Figure 3. Percentage pollen and spores diagram from Lago Cipreses, showing the position and thickness of tephras. The NAP sum is truncated to facilitate visualization of changes during CC events.

**
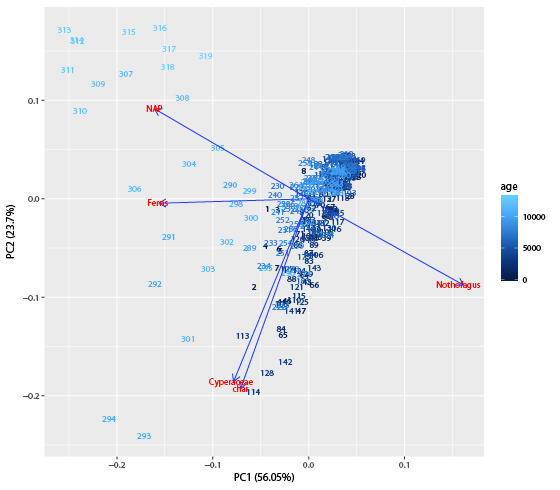
**

| scores | PC1 | PC2 |
| --- | --- | --- |
| Nothofagus | 0.547 | -0.296 |
| Cyperaceae | -0.265 | -0.627 |
| NAP | -0.543 | 0.309 |
| Ferns | -0.528 | -0.015 |
| char | -0.240 | -0.651 |

Supplementary Figure 4. PCA biplot of the entire LC record showing axes 1 and 2, and the PCA scores for the five variables under analysis. The blue colored numbers correspond to the depths of the palynological and macroscopic charcoal samples analyzed.

**
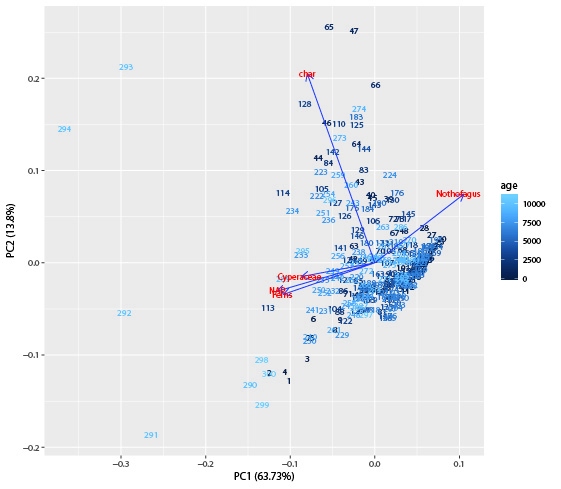
**

| scores | PC1 | PC2 |
| --- | --- | --- |
| Nothofagus | 0.471 | 0.333 |
| Cyperaceae | -0.387 | -0.067 |
| NAP | -0.517 | -0.138 |
| Ferns | -0.483 | -0.153 |
| char | -0.357 | 0.9178 |

Supplementary Figure 5. PCA biplot of the LC record younger than 10.8 ka showing axes 1 and 2, and the PCA scores for the five variables under analysis. The blue colored numbers correspond to the depths of the palynological and macroscopic charcoal samples analyzed.


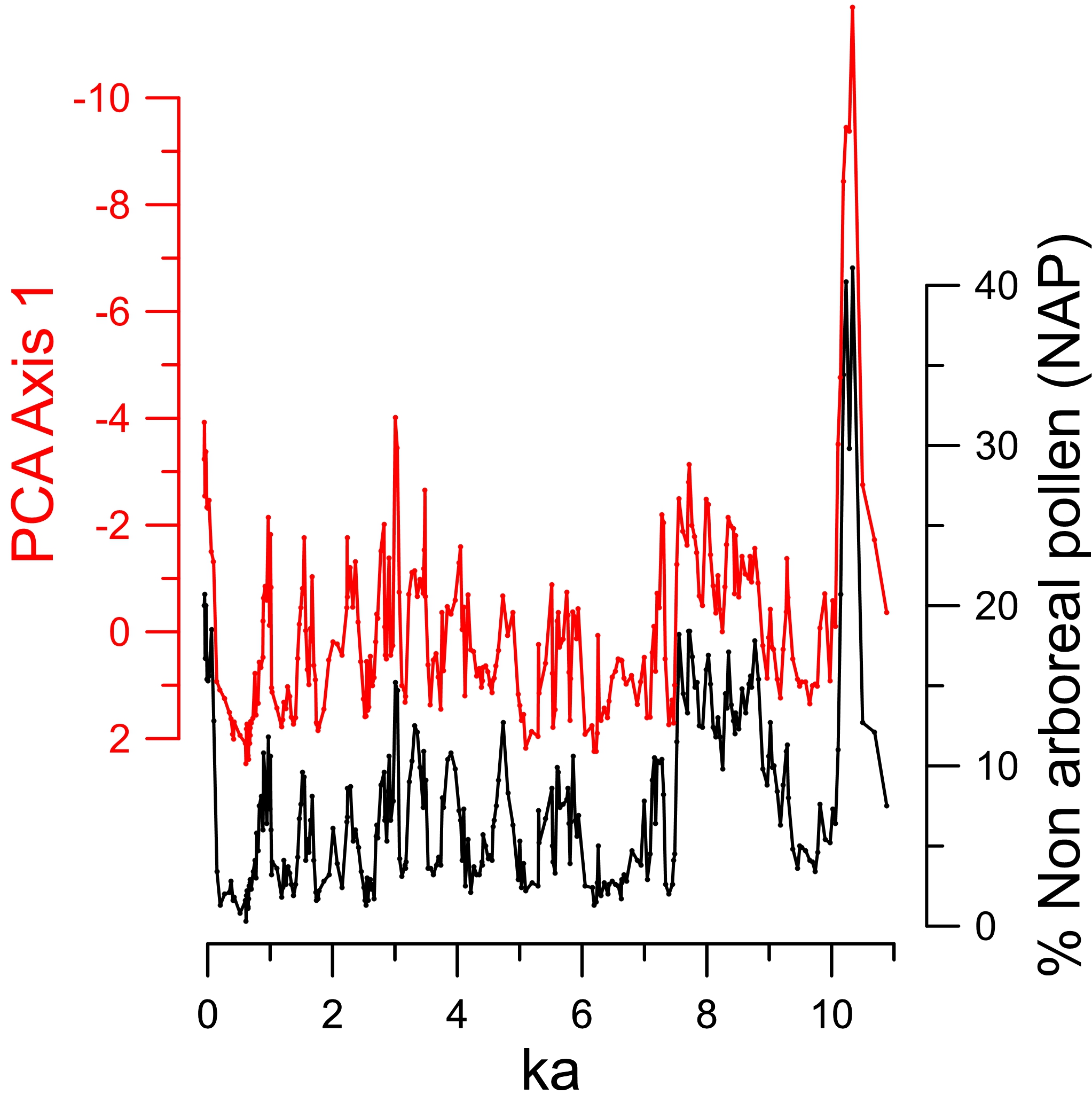


Supplementary Figure 6. Comparison of PCA Axis 1 scores (red curve) with % NAP (black curve). These results suggest that variations in NAP constitute the main source of variation in the palynology and macroscopic charcoal record in the Lago Cipreses record over the last 10,800 years.


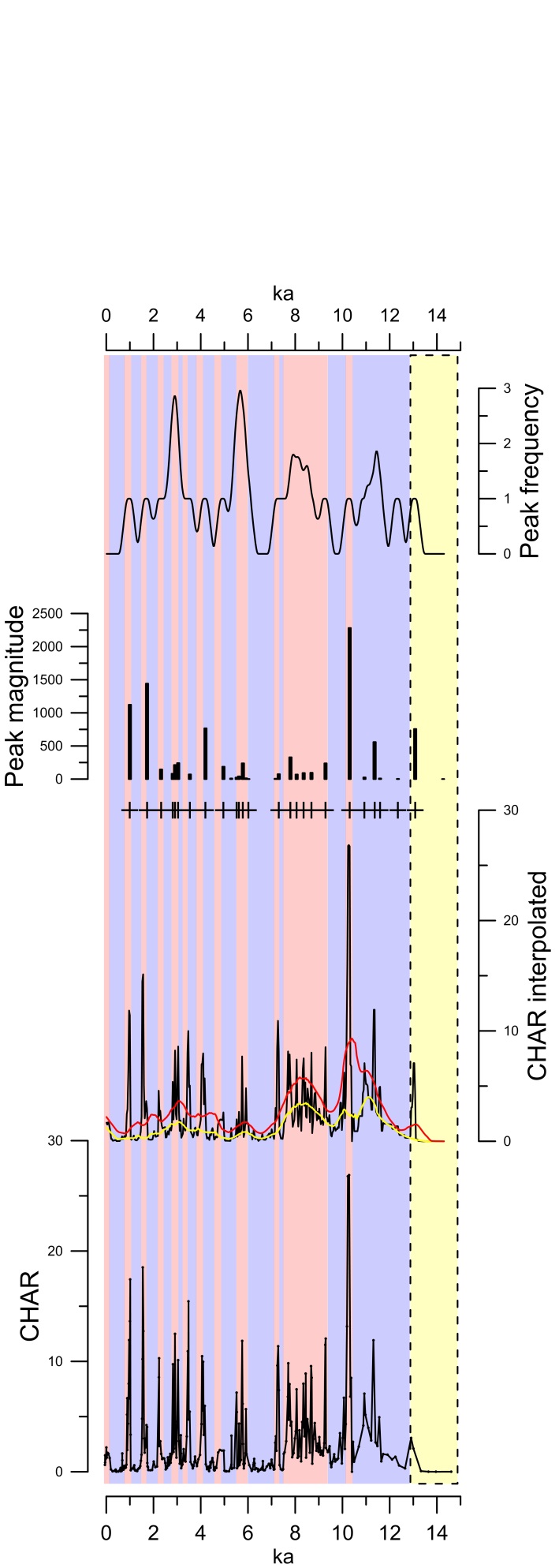


Supplementary Figure 7. Comparison of the raw CHAR with the interpolated CHAR, illustrating the close match between both, the background (yellow line) and threshold (red line) levels, the statistically significant peaks (+ symbols), peak magnitude and peak frequency.


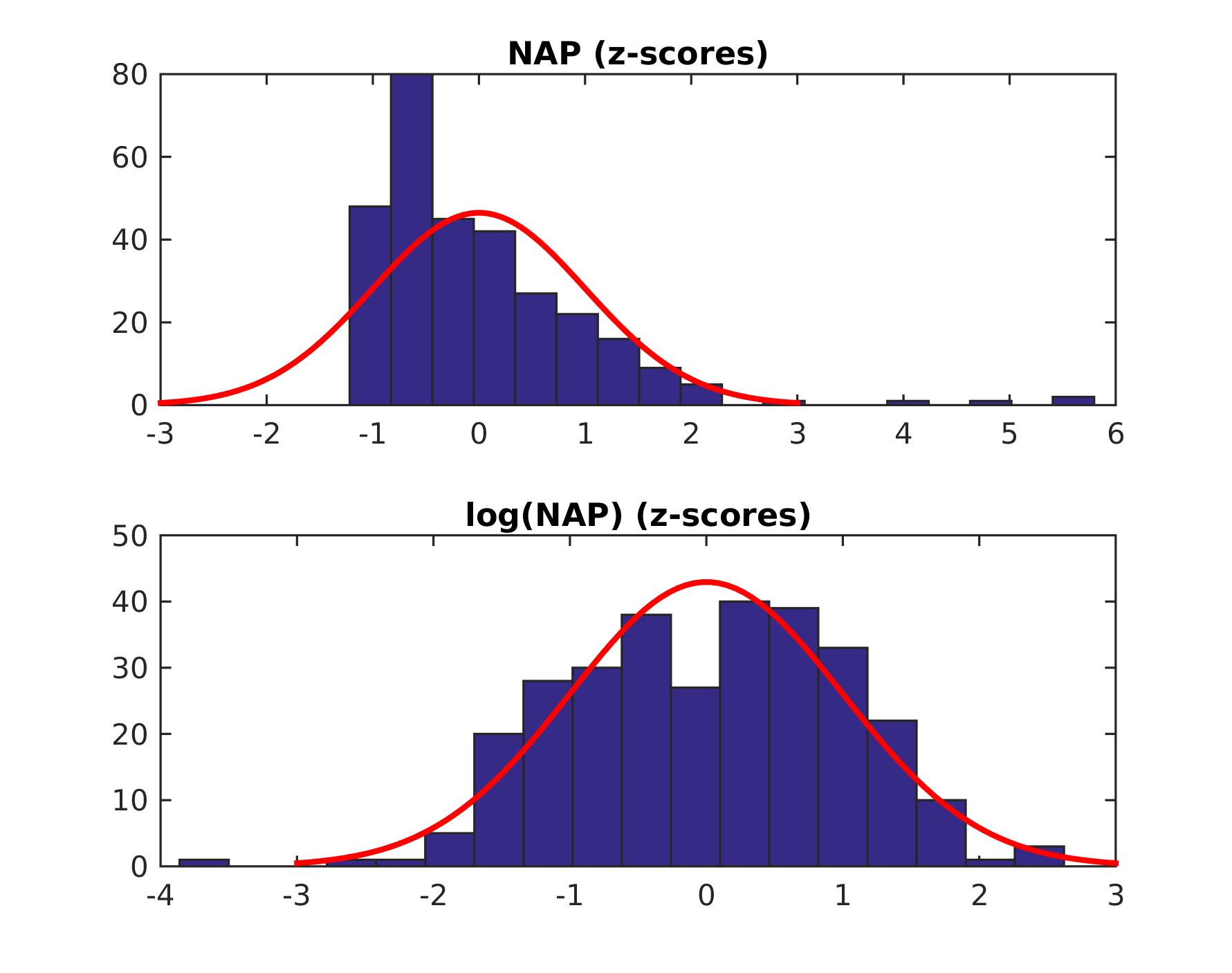


Supplementary Figure 8. Histogram of the z-scores of the LC-NAP and of the logarithmized LC-NAP data. The red line shows a fit to a Gaussian distribution.


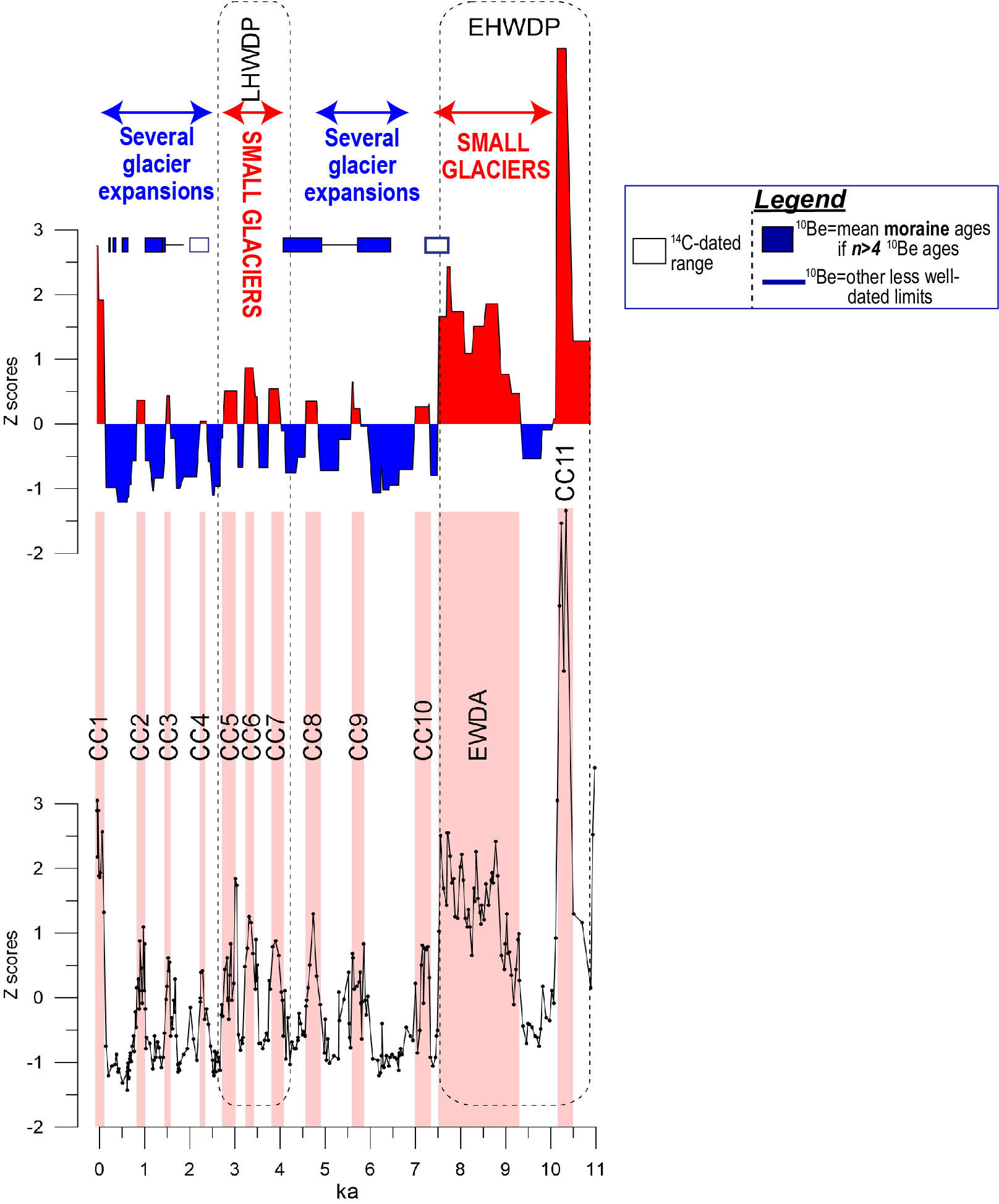


Supplementary Figure 9. Glacier history from Lago Argentino compared with the results of the regime shift detection algorithm applied to the LC NAP standardized data. Positive anomalies are shown with red filling, negative anomalies with blue filling.

| Laboratory  Code | Depth  (cm) | ^14^C yr BP  ± 1σ | lower intercept cal yr BP | upper intercept cal yr BP | median probability  cal yr BP | δ^13^C  PDB |
| --- | --- | --- | --- | --- | --- | --- |
| UCIAMS-122773 | 18 | 660±20 | 553 | 649 | 607 | -31.80 |
| UCIAMS-122774 | 31 | 835±20 | 676 | 734 | 706 | -30.17 |
| CAMS-149316 | 36 | 955±30 | 750 | 913 | 833 | -30.69 |
| CAMS-149320 | 55 | 1425±30 | 1194 | 1350 | 1296 | -32.79 |
| CAMS-149318 | 77 | 1765±30 | 1564 | 1708 | 1644 | -31.15 |
| CAMS-158326 | 97 | 2560±30 | 2473 | 2745 | 2594 | -29.99 |
| CAMS-158327 | 116 | 2945±30 | 2929 | 3163 | 3039 | -28.72 |
| UCIAMS-133081 | 145 | 3660±30 | 3835 | 4076 | 3928 | -28.72 |
| UCIAMS-133092 | 145 | 3280±30 | 3384 | 3560 | 3459 | -27.88 |
| CAMS-158468 | 171 | 4410±30 | 4853 | 5039 | 4931 | -28.78 |
| CAMS-158337 | 171 | 4450±30 | 4861 | 5270 | 4980 | -27.57 |
| UCIAMS-133089 | 205 | 5550±20 | 6220 | 6396 | 6302 | -27.20 |
| UCIAMS-133093 | 205 | 5360±20 | 5996 | 6188 | 6092 | -29.76 |
| CAMS-131268 | 233 | 6695±30 | 7463 | 7586 | 7532 | -24.81 |
| UCIAMS-133090 | 250 | 7620±25 | 8345 | 8421 | 8390 | -31.80 |
| UCIAMS-133094 | 250 | 7120±25 | 7843 | 7966 | 7901 | -30.17 |
| CAMS-158467 | 269 | 8070±35 | 8727 | 9023 | 8894 | -30.69 |
| UCIAMS-133091 | 291 | 8985±25 | 9918 | 10203 | 10,055 | -32.79 |
| UCIAMS-133095 | 291 | 6520±25 | 7315 | 7455 | 7380 | -31.15 |
| CAMS-131269 | 297 | 9435±40 | 10509 | 10730 | 10,624 | -29.99 |
| CAMS-158466 | 306 | 10055±35 | 11362 | 11764 | 11,574 | -28.72 |
| CAMS-131270 | 317 | 11275±30 | 13065 | 13195 | 13,120 | -28.72 |
| CAMS-131271 | 321 | 12465±35 | 14281 | 14959 | 14,629 | -27.88 |

Supplementary Table 1. Information on the radiocarbon dates from core PS0710. All radiocarbon dates were converted to calendar ages before present using the southern hemisphere calibration dataset (SHcal13) included for dates <11 ka and the northern hemisphere terrestrial dataset (intcal13) for dates >11 ka contained in the CALIB 7.0 software.

|  | Start  cal yr BP | End  cal yr BP | Duration  (years) |
| --- | --- | --- | --- |
| CC1 | 146 |  | 146 |
| CC2 | 1004 | 826 | 178 |
| CC3 | 1574 | 1494 | 80 |
| CC4 | 2371 | 2231 | 139 |
| CC5 | 3039 | 2775 | 264 |
| CC6 | 3494 | 3226 | 268 |
| CC7 | 3967 | 3757 | 209 |
| CC8 | 4810 | 4572 | 238 |
| CC9 | 5774 | 5603 | 171 |
| CC10 | 7280 | 6996 | 283 |
| EWDA | 9305 | 7519 | 1785 |
| CC11 | 10337 | 10145 | 192 |

Supplementary Table 2. Interpolated median probability ages for the major transitions identified in the LC record.

Supplementary references

1 Marden, C. J. *Late Quaternary glacial history of the South Patagonian Ice Field at Torres del Paine, Chile* Ph.D. thesis, University of Aberdeen, (1993).

2 Solari, M. A., Le Roux, J. P., Herve, F., Airo, A. & Calderon, M. Evolution of the Great Tehuelche Paleolake in the Torres del Paine National Park of Chilean Patagonia during the Last Glacial Maximum and Holocene. *Andean Geology* **39**, 1-21, doi:10.5027/andgeoV39N1-a01 (2012).

3 Moreno, P. I. *et al.* Southern Annular Mode-like changes in southwestern Patagonia at centennial timescales over the last three millennia. *Nature Communications* **5**, doi:10.1038/ncomms5375 (2014).

4 Blaauw, M. & Christen, J. A. Flexible Paleoclimate Age-Depth Models Using an Autoregressive Gamma Process. *Bayesian Analysis* **6**, 457-474, doi:10.1214/11-ba618 (2011).

5 Heiri, O., Lotter, A. F. & Lemcke, G. Loss on ignition as a method for estimating organic and carbonate content in sediments: reproducibility and comparability of results. *Journal of Paleolimnology* **25**, 101-110 (2001).

6 Faegri, K. & Iversen, J. *Textbook of pollen analysis*. (John Wiley & Sons, 1989).

7 Rodionov, S. N. A sequential algorithm for testing climate regime shifts. *Geophys. Res. Lett.* **31**, doi:L0920410.1029/2004gl019448 (2004).

8 Grimm, E. C. & Jacobson, G. L. Fossil-pollen evidence for abrupt climatic changes during the past 18,000 years in eastern North America. *Climate Dynamics* **6**, 179-184 (1992).

9 Higuera, P. E., Brubaker, L. B., Anderson, P. M., Hu, F. S. & Brown, T. A. Vegetation mediated the impacts of postglacial climate change on fire regimes in the south-central Brooks Range, Alaska. *Ecological Monographs* **79**, 201-219, doi:10.1890/07-2019.1 (2009).

10 Heusser, C. J. *Pollen and Spores from Chile*. (University of Arizona Press, 1971).

11 Henríquez, W. I., Moreno, P. I., Alloway, B. V. & Villarosa, G. Vegetation and climate change, fire-regime shifts and volcanic disturbance in Chiloé Continental (43°S) during the last 10,000 years. *Quaternary Science Reviews* **123**, 158-167, doi:<http://dx.doi.org/10.1016/j.quascirev.2015.06.017> (2015).

12 Jara, I. A. & Moreno, P. I. Climatic and disturbance influences on the temperate rainforests of northwestern Patagonia (40 °S) since ∼14,500 cal yr BP. *Quaternary Science Reviews* **90**, 217-228, doi:<http://dx.doi.org/10.1016/j.quascirev.2014.01.024> (2014).

13 Grinsted, A., Moore, J. C. & Jevrejeva, S. Application of the cross wavelet transform and wavelet coherence to geophysical time series. *Nonlin. Processes Geophys.* **11**, 561-566, doi:10.5194/npg-11-561-2004 (2004).

14 Strelin, J. A., Kaplan, M. R., Vandergoes, M. J., Denton, G. H. & Schaefer, J. M. Holocene glacier history of the Lago Argentino basin, Southern Patagonian Icefield. *Quaternary Science Reviews* **101**, 124-145, doi:<http://dx.doi.org/10.1016/j.quascirev.2014.06.026> (2014).

15 Aniya, M. Holocene variations of Ameghino Glacier, southern Patagonia. *Holocene* **6**, 247-252 (1996).

16 Mercer, J. H. Variations of some Patagonian glaciers since the Late-Glacial. *American Journal of Science* **266**, 91- 109 (1968).

17 Kaplan, M. R. *et al.* Patagonian and southern South Atlantic view of Holocene climate. *Quaternary Science Reviews* **141**, 112-125, doi:<http://dx.doi.org/10.1016/j.quascirev.2016.03.014> (2016).
